# American Medical Association

**Published:** 1850-07

**Authors:** 


### Part 5.—Editorial.

### ARTICLE I.

#### AMERICAN MEDICAL ASSOCIATION.

Having attended the late meeting of this annual convocation of representatives of the medical profession in the United States, at Cincinnati, although not as delegate from our readers, but of the "Chicago Medical Society," we notwithstanding feel an obligation resting upon us to give them a pretty full account of the doings of that important and distinguished body, supposing that it will be as interesting to them as anything we can lay before them at this time.

The Association met in the spacious hall of Cincinnati College, at 10½ o'clock, A. M., on the 7th of May, the President, Dr. J. C. Warren, of Boston, in the Chair, who opened the session by an address, after hearing a report of those members who had presented their credentials and registered their names, from the Committee of Arrangements. The number of delegates in attendance during the session was near three hundred.

Dr. Brainard, of Chicago, moved that members by invitation, be now proposed and received, that they may participate in the organization of the Association; upon which an animated discussion arose, and the matter was, on motion of Dr. White, of Buffalo, referred to a special committee.

On motion of Dr. Watson, of N. Y., the delegates from the several States represented were directed to select one of their number to act on a committee to nominate officers for the Association.

The committee thus chosen consisted of Drs. N. Sanborn, of N. H., E. Fowler, of R. I., W. Hooker, of Conn., C. Jewett, of Vt., E. Reynolds, of Mass., T. W. Blatchford, of N. Y., E. Fithian, of N. J., C. Morris, of Penna., J. Couper, of

Del., S. P. Smith, of Md., G. L. Corbin, of Va., W. A. Norwood, of N. C., H. R. Frost, of S. C., P. F. Eve, of Ga., W. B. Johnson, of Ala., B. Bowler, of La., N. F. Thomas, of Tenn., E. Q. Blackburn, of Ky., G. W. Boerstler, of O., A. B. Palmar, of Mich., H. G. Sexton, of Ind., J. Evans, of Ills., W. M. McPheters, of Mo., S. G. Armor, of Iowa, and J. Parker, of Wis., showing that twenty-five States were represented.

The members of the Committee of Arrangements appointed last year, and Dr. C. Caldwell, of Ky., were elected permanent members.

*Afternoon Session.*—The Committee on Publication submitted their report, with a series of resolutions making the assessment and the price of the "Transactions" the same as last year, and requiring copies of the reports of the committees to be handed to the Secretary immediately after being read, transcribed in a plain and legible hand, which were adopted.

The report of the committee on Higiene after being partly read, was referred to the Committee on Publication.

The Committee on Nominations reported as officers for the present year,

*President.*—R. D. Mussey, of Ohio.

*Vice-Presidents.*——

1st. J. B. Johnson, of Missouri.
2d. A. Lopez, of Alabama.
3d. Daniel Brainard, of Illinois.
4th. G. W. Norris, of Pennsylvania.

*Secretaries.*—A. Stillè of Pennsylvania, and H. W. De Saussure, of South Carolina.

*Treasurer.*—Isaac Hays, of Pennsylvania.

After some discussion and various motions, the report was adopted, and the nominees declared to be elected.

May 8th, 1850.

The following resolutions, offered by Dr. Bowditch, of Massachusetts, were unanimously adopted:—

*Resolved*, That the American Medical Association has learned with deep regret of the death of Prof. Harrison, their late Vice-President, and they hereby wish to express their high sense of the virtues, talents, and professional merit o their distinguished associate.

*Resolved*, That in dying, as he did, while engaged in ministering to the wants, and relieving the sufferings of his fellow-citizens, this Association recognizes in him, a noble example of professional self-sacrifice.

*Resolved*, That the warmest sympathies of this Association are hereby most respectfully tendered to the family of their honored and deceased associate.

On motion of Dr. Stille, it was

*Resolved*, That a properly authenticated copy of the resolutions be transmitted to the family of Dr. Harrison.

The newly elected officers were conducted to their stations by an appropriate committee, when the President, in a few brief and pertinent remarks, returned thanks for the honor conferred upon him.

The committee on members by invitation, reported, and the following resolution was adopted:—

*Resolved*, That all those gentlemen who have been nominated to the Association be admitted as members by invitation.

Dr. Hooker, of Conn., moved the following:—

*Resolved*, That the section in the Constitution relating to members by invitation be repealed; which lies over, according to rule, to the next meeting of the Association.

Dr. Blatchford, of New York, presented the report of the committee on education, which he requested might be read by the Secretary, as the chairman of the committee was absent. The Secretary read the report.

Dr. Blatchford offered the following preamble and resolutions, prefacing them with the remarks, that although a member of the committee, he had never seen the report until late on the preceding evening, and that he dissented altogether from the opinions it expressed.

*Whereas*, This Association has learnt through its several committees, appointed from year to year to examine into the state of medical education in our country, that many of the medical colleges, invested by law with the power of granting degrees, still continue a system of instruction which we cannot but regard as defective, both in the time allotted to the delivery of lectures, in the attention paid to practical anatomy, in the facilities afforded for clinical instruction, and in the low standard of the requirements for a degree; therefore,

*Resolved*, That this Association reiterates its former recommendations upon these points, and would urge upon the medical colleges to continue their efforts to elevate the standard of medical eduaation, by adopting such changes in their courses of instruction as shall satisfy the just and reasonable desires of the profession.

*Resolved*, That the thanks of the American Medical Association are due to the Faculties of the University of Pennsylvania and of the College of Physicians and Surgeons of New York, and all other institutions which may have conformed to our recommendations, for their prompt response to the recommendations of the Association for the improvement of medical education.

Dr. Roberts, also a member of the committee, dissented from the report, and said he had not seen it until the preceding evening.

Dr. Stille stated that the Colleges of Pharmacy of New York and Philadelphia were in active operation, as a correction to the report.

Dr. Parish, of Philadelphia, addressed the association in an able manner and at length, in opposition to the vews of the report.

After the offering and withdrawal of several motions, it was on motion of Dr. Stille

*Resolved*, That the report of the chairman of the committee on medical education be recommitted for correction as to matters of fact, and then handed to the committee of publication.

*Resolved*, That the resolutions of Dr. Blatchford be made the special order for the meeting of this afternoon.

The nominating committe were instructed to report committees and select a site for the next meeting of the Association.

*Afternoon Session.*—Dr. Johnson, Vice-President, in the chair. The consideration of Dr. Blatchford's resolutions being the order of the day.

Dr. Annan, of Ky., had in the forenoon observed that he had been a member of the first convention, and was there humiliated by the silence of the professors. The cry was "*pecavi*," "*Ora pro nobis.*" He would defend them from the ungrounded charges made. He now proceeded and said:—

The recommendations of the Association could not be carried out. The material did not exist. Young men with the requisite qualifications, and in numbers to supply the demand, could not be found who would enter the schools. We must take what we have or none. Even in the great cities this was the case. The gentlemen, members of the Association, even, who received these men into their offices, took their fees, and then sent them to the schools, were primarily to blame for their inadequate qualifications. Furthermore, he denied the vaunted superiority of graduates of those schools which had complied with the recommendations, over those of schools who had not. The reason why the western schools had not complied with the recommendations was, that they had not deemed them practicable, however reasonable they might seem. They deemed a course of lectures, of four months' duration, sufficient, if properly condensed. In the second place, students had not the means even for attending four months; and, as medicine was free, they would go where they should not exceed their means, or they would be driven to practise without the advantage of hearing a course of lectures. Unquestionably, practical anatomy *was* important; but this was pursued as far as the schools could furnish the means, which, in point of fact, considering the relative numbers of the classes, were greater in Kentucky than in Philadelphia. The clinical instruction of the Philadelphia schools, given to three or four hundred students, carried once a week to the hospitals in omnibusses, he regarded as insignificant and comparatively worthless. He wondered that the Philadelphia professors did not laugh at the figure they exhibited in their dispensary *exhibitions*.

Dr. Miller, of Louisville, moved to add to the first resolution the words, "and urges upon the lay members of the Association to *begin* the work of improvement, by their efforts to raise the qualifications of candidates for matriculation."

While he was disposed, experienced as he was, in good spirit to receive the homilies that were read to him, he could not, as the discussion had turned, refrain from asking, how the *practitioners* performed *their* duty. In point of fact, not one in fifty in the United States bestowed any adequate attention upon the business of instruction. The mass utterly neglected young men, merely placing books in their hands, indicating no order of reading or of study, and leaving them to pursue their own course. If this was not a *caricature*, with what face, then, could they spur on the professional corps? They (the Professors) wept over the inadequate preparation of young men to receive professional training. They could not *enforce* the recommendations of the Association. It was absurd for them to set up as school-masters. They were willing to do all in their power. Let the Associciation do *its* part. The duty was reciprocal.

Dr. Davis commenced by an allusion to Dr. Annan's complaints that the colleges had been unjustly accused, and instead of defending themselves had meekly cried, "Peccavi," &c. He reminded Dr. A. that if the colleges had been accused in any of the reports to this Association, it was certainly the fault of their Professors, for they had filled most of the offices and headed five-sixths of the standing committees ever since the organization of the Association; a fact, which in itself showed that the supposed war upon colleges was far more imaginary than real. The subject under discussion, however, was one of great interest, both to the profession and the community. He ridiculed the idea that four months was long enough to give or hear a satisfactory course of lectures on all the branches of medical scienee, as they should be taught at the present day. Let the absolute number of medical men be too great or too small, one thing is certain, that *too small* a number are properly educated and qualified to practice their profession. This Association had again and again recommended better preliminary education, longer periods of college instruction, and especially better practical or clinical advantages. And all this is right, but how shall it be accomplished? How shall a greater number of students be induced to study longer, and qualify themselves more thoroughly?

It is objected that the students cannot afford to study longer, and especially to attend a longer period of college instruction. It is true that very few of the wealthy are attracted either to the study or practice of medicine, and consequently that a large majority of medical students are much cotrolled in their choice of educational means, by pecuniary considerations. If it is desirable then, to induce the great majority of students to study longer, to avail themselves of true clinical or hospital instruction, and to qualify themselves in every sense to do honor to the profession and good to mankind, the present order of things in reference to colleges must be reversed; and those institutions which are so located as to possess all the requisite advantages for giving clinical as well mere oral instruction must so arrange their *fees* as to destroy the advantage now almost universally possessed by institutions so located as to be entirely destitute of even the semblance of an hospital within their reach. That pretended professional dignity which induces the only colleges so located as to have the required means of instruction within their reach, to charge such *ticket fees* as almost neeessarily to exclude from their halls one-half of the whole number of medical students, must give place to a system of charges better adapted to the pecuniary condition of those seeking knowledge.

If it is objected that by cheapening medical education, we only induce a greater number to enter upon its study and thereby increase the already crowded state of the profession, we reply, in the first place, that so long as the laws of the States universally permit every man to practice medicine who wishes, whether he has ever seen the inside of a college or not, the objection has no force; and in the second place, that the number admitted into the profession should be limited by elevating the standard of requirements, instead of merely taxing the student's pockets. He said all present had one object in view, viz: the elevation of the profession and the extension of its usefulness; and he trusted that no mere local feelings or personal interests would ever sway our action. And most of all should he regret to see the Association show the least disposition to take a single step backward in the great work it had undertaken.

Dr. Baker, of Cincinnati, thought circulars, holding out special advantages of one school over another, should be condemned. The code of ethics should be extended to colleges as well as individuals.

Dr. Wright, of Cincinnati, thought it strange that there should be so much discussion on matters so plain—was in favor of lengthening the term in all medical schools except the Med. College of Ohio. After some fluent and pungent remarks, of a personal nature, introduced in very bad taste and to the great annoyance of the Association, he was called to order.

Dr. Rives, of Cincinnati, followed, but in endeavoring to answer the charges of Dr. Wright, against the Medical College of Ohio, was soon found out of order.

Dr. D. W. Yandall, of Ky., made a labored effort to impress upon the Association a full sense of the deficiencies of clincal instruction in the schools of the West. His effort, though in some respects creditable, was too strong in denunciation. It served as an offset to Prof. Annan's remarks against schools at the East.

Dr. McPheters, of Mo., defended the medical schools of St. Louis, in reference to clinical teaching.

May 9th.

The consideration of Dr. Blatchford's resolutions was resumed.

On motion of Dr. Drake, the speakers were restricted to ten minutes each, and no one allowed to speak more than twice on the same resolution.

After some confusion, the Association went into Committee of the Whole, Dr. Knight, of Connecticut, in the chair.

The forenoon was spent in the discussion. We are sorry our limits will not allow a full report of it.

Drs. Morris, of Phila., Miller, of Louisville, Storer, of Boston, Lawson and Drake, of Cincinnati, Mitchell, of Philadelphia, Gilman, of N. Y., Ware, of Boston, Kerfoot, of Pa., Powell and Raphael, of Ky., Little, of Pa., Stille, of Pa., and Annan, of Ky., took part in the debate, and many amendments and substitutes were offered.

After voting to strike out the names of particular colleges, the following, offered as a substitute for the whole matter before the committee, by Dr. Morris, was adopted:

*Resolved*, That the Association re-affirms its recommendations in regard to medical education, and urges that private instructors should receive into their offices, as students, only those qualified by previous instruction to pursue the study of medicine.

The committee then rose, and the Chairman reported accordingly. The report was accepted, and the whole matter was referred to the committee on medical education for the ensuing year.

Dr. Lopez, Vice-President, asked and obtained leave to read a protest on behalf of the profession of Alabama, against certain statements in regard to legal requirements, and unjust to the medical faculty of that State, made in the report on Medical Education, of 1849, which protest was accepted and referred to the Committee on Publication.

*Afternoon Session.*—Dr. Mitchell, Chairman, read a lengthy and well written report on Practical Medicine, principally devoted to the subjects of Cholera and Scarlatina. It was referred to the Committee on Publication, after a general expression that the acceptance of the report did not imply the adoption of the doctrines advanced by it.

The Committee on Nominations reported the Standing Committees for the ensuing year. We give the Chairman of each:—

On Medical Sciences, Dr. Bennett Dowler, of New Orleans, La.

On Practical Medicine, Dr. Austin Flint, Buffalo, N. Y.

On Surgery, Dr. Paul F. Eve, of Augusta, Georgia.

On Obstetrics, Dr. C. Humphreys Storer, of Boston, Mass.

On Medical Education, Dr. Worthington Hooker, of Norwich, Conn.

On Medical Literature, Dr. Thomas Reyburn, of St. Louis, Mo.

On Publication, Dr. Isaac Hays, of Philadelphia, Pa.

Committee of Arrangements, Dr. H. R. Frost, f Charleston, S. C.

The committee also recommended the city of Charleston, S. C., for the place of meeting, on the first Tuesday of May, 1851. The nominations were confirmed.

The Secretary read a letter addressed to Dr. Evans, from Dr. Prioleau, Chairman of the Committee of Obstetrics, who was unexpectedly prevented from attending the meeting, giving as a reason for not preparing a formal report, that there had come to his knowledge no important American improvement in that department of medicine during the year.

Dr. Evans, of Chicago, presented a paper on the "*Obstetrical Extractor*," invented by himself, and demonstrated upon the manakin, the mode of its application in practice.

The paper, with the letter from Dr. Prioleau, was referred to the appropriate committee, with discretionary power as to their publication.

Dr. Drake, Chairman of the Committee of Arrangements introduced a paper from Dr. N. S. Davis, of Chicago, upon the question, "Has the cerebellum any special connection with the sexual propensity, or function of generation?"

It was read by its author, and referred to the Committee on Publication.

An entertainment was given in the evening, at Masonic Hall, by the regular profession of Cincinnati. It was most sumptuous, and to the credit of the Committee of Arrangements, they did not furnish intoxicating drinks for beverage.

May 10th.

Dr. Johnson, Vice-President, in the Chair.

Dr. Parsons, Chairman of Committee on Medical Sciences, presented the report which was received and referred to the Committee on Publication.

Dr. Huston, Chairman, read the report of the Committee on Spurious and Adulterated Drugs, which was referred to the Committee on Publication.

Resolutions accompanying the report were adopted, and a committee of one from each State represented was appointed to collect information in reference to spurious and adulterated drugs, to report at the next meeting of the Association.

Dr. C. Caldwell was requested to prepare a paper showing how far he thinks phrenology and mesmerism are founded in truth—also how far vital or animal chemistry is entitled to the appellation of a science.

The Committee on Nominations reported committees on Indigenous Medical Botany, Dr. A. Clapp, of New Albany, Ia., Chairman, and on Hygiene, Dr. James Moultrie, of Charleston, South Carolina, Chairman, which report was confirmed.

On motion of Dr. Blatchford, a special committee on pharmacy and the adulteration of drugs was appointed by the President, Dr. T. O. Edwards, of Ohio, to be Chairman.

On motion of Dr. Morris, of Pa., it was

*Resolved.* That it is with great satisfaction the members of this Association have observed the establishment of drug stores, in which neither patent medicine, nostrums, nor other articles, by which the artful and designing impose on the ignorant and credulous, are exposed for sale; and that the Association recommends to its members to exert their influence in their respective spheres of action to encourage similar efforts in other places.

On motion of Dr. Phelps, of N. Y., the subject of clerical encouragement of quackery was referred to the Committee on Hygiene.

Dr. Hooker, of Connecticut, offered resolutions in reference secret modes of practice by physicians, condemning them, which were adopted.

Dr. Lawson offered a resolution condemning the advertisement of nostrums by Medical Journals—adopted.

*Afternoon Session.*---Dr. Miller, of Ky., moved a suspension of the rules, and offered the following which was adopted:

*Whereas*, clinical instruction in medicine and surgery is now generally acknowledged to be essential to the proper qualification of students for the practice of those branches of our profession, and whereas, it must be admitted that clinical instruction in midwifery would be equally valuable, therefore,

*Resolved*, That the Committee on Medical Education be instructed to inquire whether any practicable scheme can be devised to render instruction in midwifery more practical than it has hitherto been in the medical schools of the United States, and report at the next meeting of this Association.

Dr. Stille, Chairman, read the report on Medical Literature, which was accepted and referred to the Committee on Publication.

A series of resolutions accompanying it were considered *seriatim*, and adopted.

They recommend the patronage of medical periodicals; forming medical reading clubs; offering pecuniary rewards for the best memoirs; and that a committee be appointed to award a premium of $100, to be raised by voluntary contribution for "the best *experimental* essay on a subject connected either with physiology or medical chemistry.

Dr. F. G. Smith, of Philadelphia, is Chairman of said committee.

The Committee on International Copy-Right Law reported a memorial to Congress, which was ordered to be signed by the officers and forwaided.

On motion of Dr. Gross, of Ky., it was resolved that the Association recommend to colleges to adopt native productions as text-books for their pupils, and that the editing of English works by Americans be condemned.

On motion of Dr. Roberts, of Md., a committee of three on unfinished business was appointed. Dr. Roberts was appointed Chairman.

Dr. Drake, of Cincinnati, offered an amendment to the Constitutien, providing that candidates for membership, by invitation, be proposed in writing by five members, and that all permananent members be allowed to vote.

Dr. McGuire, of Va, offered resolutions in reference to the medical staff of the army and navy of the United States, which were unanimously adopted.

On motion of Dr. Bowditch, of Boston, the committee on Medical Education were instructed to inquire if any means could be adopted to secure more thorough instruction in practical chemistry.

The Secretary presented the report of the Committee on Indigenous Medical Botany; a report on Vital Statististics of New Orleans, by Dr. J. C. Simmonds; Biographical Notices of Deceased Physicians, by Dr. Williams, of Mass., which were referred to the Committee on Publication, and a catalogue of Indigenous Medical Botany, by Dr. Bigelow, of Ohio, which was referred to the Committee on Medical Botany.

On motion of Dr. Flint, of N. Y., it was resolved that the MSS. work of the late lamented Dr. Forry be referred to the Committee on Publication, to be published if they deem advisable.

Dr. Gross, of Ky., moved the appointment of a committee to consider the propriety of the establishment of schools of veterinary medicine and surgery; adopted. Dr. Gross was appointed Chairman of said committee.

Dr. Kreider, of Ohio, presented a protest from the Fairfield Co. Medical Institute against the vending of spurious drugs; referred to the special committee on the subject.

Dr. Mead, of Ills., offered resolutions instructing the committee on Medical Education to enquire into the propriety of abolishing the rule that allows four years practice in lieu of one course of lectures, and of making college fees uniform, varying only between the North and South. Adopted.

After the passage of votes of thanks to the Committee of Arrangements and to the physicians of Cincinnati for the kindness and attention shown, and to the Trustees of Cincinnati College for the use of their spacious Hall, the Association adjourned *sine die.*

Many pleasant acquaintances are formed, and much harmony of feeling and action secured by these convocations, and we hope the year is far in the future that shall not record a meeting of the American Medical Association.

We are indebted to the Medical News and the Boston Medcal and Surgical Journal for mnch of the material for making up this abstract. It may be relied upon as being generally correct. E.

